# Evaluation of the in vitro human skin percutaneous absorption of ketoprofen in topical anhydrous and aqueous gels

**DOI:** 10.1111/srt.13589

**Published:** 2024-02-23

**Authors:** Kendice Ip, Guiyun Song, Daniel Banov, August S. Bassani, Yi Liu, Hui Song, Benigno C. Valdez

**Affiliations:** ^1^ Professional Compounding Centers of America (PCCA) Houston Texas USA; ^2^ Department of Stem Cell Transplantation and Cellular Therapy The University of Texas MD, Anderson Cancer Center Houston Texas USA

**Keywords:** anhydrous gel, extemporaneously compounded formulations, permeation enhancers, skin, topical

## Abstract

**Background:**

Ketoprofen is a nonsteroidal anti‐inflammatory drug used for the treatment of acute and chronic pain associated with inflammatory conditions. This study aims to evaluate the in vitro percutaneous absorption of ketoprofen 10% formulated in proprietary anhydrous and aqueous gels using the Franz skin finite dose model.

**Materials and Methods:**

The anhydrous gel was initially characterized for cytotoxicity using EpiDerm skin tissue model by cell proliferation assay and Western blot analysis. The Ultra Performance Liquid Chromatography method for measuring ketoprofen was validated and the stability of ketoprofen 10% in the anhydrous gel formulation was evaluated at 5°C and 25°C for 181 days. The percutaneous absorption of ketoprofen was determined using donated human skin. The tissue sections were mounted within Franz diffusion cells. A variable finite dose of each ketoprofen formulation in either anhydrous or aqueous gel was applied to the skin sections and receptor solutions were collected at various time points.

**Results:**

Cell proliferation assay showed minimal cell death when EpiDerm skin tissue was exposed to the anhydrous gel for 24 h; the levels of protein markers of cell proliferation were not affected after 17‐h exposure. Ketoprofen was stable in the anhydrous gel when stored at 5°C and 25°C. When compounded in the anhydrous and aqueous gels, ketoprofen had mean flux rate of 2.22 and 2.50 μg/cm^2^/h, respectively, after 48 h. The drug was distributed to the epidermis and dermis sections of the skin. Both the anhydrous and aqueous gels facilitated the percutaneous absorption of ketoprofen without statistically significant differences.

**Conclusion:**

The anhydrous gel can be used as a base to facilitate the transdermal delivery of ketoprofen. Although the anhydrous and aqueous gels can deliver a similar amount of ketoprofen, the anhydrous gel (water activity below 0.6) allows for extended default beyond‐use‐date of compounding preparations.

## 1 INTRODUCTION

Ketoprofen belongs to the propionic acid class of nonsteroidal anti‐inflammatory drugs (NSAID's) which present anti‐inflammatory, analgesic, and antipyretic activity. It inhibits the synthesis of prostaglandins by acting as a reversible inhibitor of the cyclooxygenases.^1^ Its oral administration is used for the treatment of acute and chronic pain conditions associated with inflammatory reactions and it is commonly prescribed for arthritis‐related pain.^2^ This route of administration, however, is associated with serious gastrointestinal (GI) adverse effects including irritations, ulcerations, bleeding, and dyspepsia.

To minimize systemic effects associated with oral ketoprofen, its transdermal delivery alternative route of administration has become increasingly popular. It is applied as gels, creams, or sprays onto the skin surface where ketoprofen is expected to penetrate through the skin, into the underlying tissue layers and systemic circulation. Transdermal delivery of ketoprofen is noninvasive and painless, convenient to administer, avoids contact with the GI organs, and prevents first‐pass metabolism by the liver.^3^ This approach may result in predictable pharmacokinetics and potentially improved bioavailability of ketoprofen.^3^, ^4^

By considering the influence of the physicochemical characteristics and pharmacokinetic properties of ketoprofen and other NSAID's, Beetge et al.^5^ showed that ketoprofen has better transdermal bioavailability than naproxen, indomethacin, and ibuprofen, which may be attributed to its lower log *p* value (partition coefficient) and its hydrophilic properties. This study suggested that ketoprofen is an appropriate candidate for transdermal delivery. In fact, the topical formulations of ketoprofen in the compounding bases Pluronic Lecithin Organogel (PLO) and Lipoderm have been characterized. Although both gels facilitated the percutaneous absorption of ketoprofen across human cadaver trunk skin, the Lipoderm formulation showed faster rate and higher mean total absorption of ketoprofen than when in PLO.^6^ Lipoderm, however, is an aqueous compounding base, which may provide a shorter default beyond‐use date (BUD) for active pharmaceutical ingredients (API). This concern underscores the importance of finding an alternative base that provides a longer default BUD.

The present study was performed to determine if a proprietary anhydrous gel (PermE8) is a good candidate for the transdermal delivery of ketoprofen, and to confirm its longer default BUD.

## 2 MATERIALS AND METHODS

### 2.1 Composition of formulations

Table 1 shows the composition of the compounded topical formulations used in this study which contained ketoprofen USP 10% in either the anhydrous gel or the aqueous gel.

**TABLE 1:** Test formula of 10% ketoprofen in anhydrous and aqueous gels.

**Ketoprofen 10% Topical Anhydrous Gel**	
100 Gm	
Ketoprofen USP, PCCA Special Micronized	10 g
Propylene Glycol USP	10 g
Base, PCCA Anhydrous Gel	80 g
**Ketoprofen 10% Topical Aqueous Gel**	
100 Gm	
Ketoprofen USP, PCCA Special Micronized	10 g
Propylene Glycol USP	10 g
Base, PCCA Aqueous Gel	80 g

### 2.2 Determination of safety of the base

#### 2.2.1 Cytotoxicity assay

The European Union Reference Laboratory (UELR ECVAM) has recommended the use of the human EpiDerm system (EPI‐200) as an alternative to the commonly used Draize test.^6^, ^7^ The model, purchased from MatTek (Ashland, MA), contained normal human‐derived epidermal keratinocytes. The three‐dimensional system was maintained with the special culture media provided by the manufacturer. The anhydrous gel (100 μL) was added onto the EpiDerm tissues and kept at 37°C for 1, 4, 17, and 24 h, and the dosing materials were removed, and tissues were analyzed for cell viability. Triton X‐100 1% solution was used as a positive control. The experiments were done in duplicate.

The MTT assay was used to determine cell viability as previously described.^8^ It measured the enzymatic reduction of 3‐[4,5‐dimethylthiazol‐2‐yl]2,5‐diphenyltetrazolium bromide (yellow solution purchased from MatTek) to formazan crystals (purple). Briefly, the media and dosing solution were removed from all wells of the EpiDerm tissue and the MTT solution was added to the basal side of the tissues and incubated at 37°C for 3 h. The purple formazan crystals produced at the apical and basal sides of the tissues were dissolved using the extractant provided by MatTek. A plate reader was used to determine the absorbance at 570 nm and using 650 nm as a reference. The relative cell viability was calculated relative to the untreated control.

#### 2.2.2 Protein analysis by western blot

EpiDerm tissues were exposed to the anhydrous gel or Triton X‐100 for 17 h. A Pellet Mixer was used to homogenize the treated tissues for approximately 1 min on ice and centrifuged at 14,000 rpm for 10 min at 4°C. Total protein concentrations in the tissue lysates were determined using a BCA Protein Assay kit (Thermo Fisher Scientific, Rockford, IL) as previously described.^9^ The samples then underwent sodium dodecyl sulfate polyacrylamide gel electrophoresis (SDS‐PAGE) to resolve proteins, which were transferred to nitrocellulose membranes (Bio‐Rad, Hercules, CA). To block the membranes, 3.5% nonfat milk in phosphate‐buffered saline was used and left for 1 h at room temperature. Specific primary antibodies were incubated overnight at 4°C, washed, and incubated with HRP‐conjugated secondary antibodies for 1 h at room temperature. Protein bands were detected using Immobilon Western Chemiluminescent horseradish peroxidase substrate (Millipore‐Sigma, St Louis, MO). The β‐actin protein was used as an internal control.

### 2.3 In vitro permeation test of ketoprofen compounded topical formulations

#### 2.3.1 Preparation of skin samples

The percutaneous absorption of ketoprofen was measured using human cadaver abdomen skin tissue from three Caucasian, female donors (Table 2). Full‐thickness skin samples were purchased from Genoskin (Salem, MA), which obtained informed consent from the donors and the ethical tissue collection was approved by the French Minister of Research (No. AC‐2022‐4863). Before using, cryopreserved skin tissues were defrosted and kept in a diffusion medium for at least 30 min at 32°C and inspected for cuts, holes, or signs of skin disease. To keep the integrity of the samples, unused skin tissues were not re‐frozen. For each compounded formula, a total of three donated skin tissues were used in three replicates.

**TABLE 2:** Donor demographics and skin integrity results.

Donor	Lot #	Age	Race	Sex	Integrity test results^a^ (kΩ)
1	20180713.02	35	Caucasian	Female	52.07 ± 10.11
2	20180627.02	22	Caucasian	Female	33.75 ± 9.36
3	20190115	46	Caucasian	Female	19.10 ± 3.70

^a^ Results are reported as electrical resistance in kΩ; acceptance >4 kΩ for dosed skin section.

#### 2.3.2 Permeation analysis using Franz cell system

Each skin sample was cut into small sections to fit on the Franz diffusion cell chamber (PermeGear, Hellertown, PA). The physiological diffusion medium was added to the receptor chamber and the integrity of the skin sections was assessed using a Precision LCR meter, set at low voltage alternating current. The skin electrical resistance was measured and samples that showed electrical resistance <4 kΩ were rejected; this criterion was based on published data^10^ which showed that 4.0 kΩ corresponded to a tritiated water permeability coefficient of 4.5 × 10^−3^ cm/h.^11^ Approximately 10 mg/cm^2^ of the compounded formulation was applied on each skin sample using a positive displacement pipette. A pellet pestle was used to spread the product across the skin surface. The integrity of the skin was maintained using the receptor solution as diffusion medium (HBSS #14175‐079, 25 mM HEPES, #15630‐080 and 50 μg/mL Gentamicin, #15750‐060, Gibco), which was stirred magnetically at 600 rpm. The water jacket temperature was maintained at 32 ± 0.5°C. For analysis, 1 mL receptor solution was obtained after 8, 24, 30, and 48 h of adding the formulations.

#### 2.3.3 Extraction of ketoprofen

Following 48 h, by the end of the diffusion process, the skin samples were washed with 50% ethanol solution, and the wash solution was centrifuged at 6000 rpm for 10 min. The supernatant was transferred into an UPLC (Ultra Performance Liquid Chromatography) vial for analysis. Then the skin samples were split into epidermis and dermis, cut into small pieces, and transferred to a separate test tube for overnight extraction with 50% ethanol.

#### 2.3.4 Quantification of ketoprofen

The quantification of ketoprofen was performed by UPLC using a reverse phase, gradient chromatography with mobile phase A for purified water, and mobile phase B for 0.1% formic acid in acetonitrile. The 1.7 μm Acquity UPLC BEH C18 2.1 mm × 50 mm column was used. The column was heated to 50°C and samples were kept at 8°C. The flow rate was set at 0.8 mL/min. The composition of the mobile phase began with 85% A and 15% B, gradually changed to 55% A and 45% B from 0 to 3 min. At 3.1 min, the gradient returned to initial composition of 85% A and 15% B and remained the same until the end of the run at 4 min. A total of six calibration standards ranging from 0.05 to 100 μg/mL were prepared to quantitate the amount of ketoprofen in sample solutions collected in the permeation test.

#### 2.3.5 Statistical analysis

Results are presented as the mean ± standard deviation of three independent experiments and statistical analysis was performed using Analysis ToolPak in 2016 Excel. *p* values less than 0.05, determined using a two‐way ANOVA, were considered statistically significant.

### 2.4 Stability study of ketoprofen compounded topical formulations

#### 2.4.1 Validation of the method

The UPLC assay method was validated by evaluating the method with regards to system suitability, linearity, accuracy, precision, robustness, solution stability, and specificity as recommended in the ICH “Harmonised Tripartide Guideline: Validation of Analytical Procedures: Text and Methodology Q2(R1)” and USP General Chapter: <1225 > Validation of Compendial Procedures. The compounded formulation and placebo were subjected to forced degradation conditions using heat (60°C, 14 d), oxidation (20% H_2_O_2_, 7 d), acid (0.1 N HCl, 7 d), and base (0.1 N NaOH, 7 d). Table 3 shows the methods, results, and acceptance criteria used for the validation.

**TABLE 3:** Assay method validation parameters, corresponding methods, acceptance criteria, and results.

Parameter	Methods	Acceptance criteria	Results
System suitability	A reference standard solution of ketoprofen at 100% assay level was prepared and analyzed 5 times.	RSD ≤ 1.0%	RSD = 0.2%
		Tailing factor ≤ 2.0	Tailing factor = 1.10
		Column Efficiency ≥ 2000	Column Efficiency = 14,814.6
Linearity	The stock solution of ketoprofen was diluted to five concentrations (80%, 90%, 100%, 110%, and 120% of assay level). Each solution was injected in triplicate to generate a calibration curve.	R^2^ ≥ 0.995	Regression line: y = 5158x–800.61, R^2^=0.9996
		y‐intercept ≤ 1.2% of target concentration	y‐intercept = 0.25%
		Maximum residual ≤ 1.2%	Maximum residual = 0.44%
Accuracy	Spiked placebo at 80%, 100% and 120% of the assay level was prepared in triplicate. Each solution was injected in triplicate, and quantitated against a 5‐point calibration curve.	98.0% ≤ Recovery ≤ 102.0%	Recovery at 80% = 101.71%
			Recovery at 100% = 100.92%
			Recovery at 120% = 101.12%

Precision (Repeatability)	Analysis of the data in the Accuracy determination at 80%, 100%, and 120% of the assay level in triplicate.	RSD ≤ 2.0%	RSD at 80% = 0.2%
			RSD at 100% = 0.9%
			RSD at 120% = 0.4%
Precision (Intermediate)	Prepared six determinations of standard solution at 100% target concentration on two different days and two different UPLC instruments.	RSD ≤ 5.0%	RSD from day 1, UPLC 1 = 0.26%
			RSD from day 2, UPLC 2 = 1.01%

Robustness	Determined with variations in column temperature, organic mobile phase content, and flow rate using spiked placebo	RSD ≤ 2.0%	Column temperature ± 2°C
		Tailing factor ≤ 2.0	Organic mobile phase ± 8%
		Column efficiency ≥ 2000	Flow rate ± 1%
		Resolution ≥ 2.0	
Solution stability	Inter‐day prepared standard solution and spiked placebo at target concentration were analyzed.	RSD ≤ 2.0%	Stability (days) = 4
			RSD from standard = 0.40%
			RSD from spiked placebo = 0.55%
Specificity	Placebo and sample were analyzed to detect degradation under stressed conditions (forced degradation studies)	No chromatogram interference	Conditions: Thermal, oxidation, acid, and base
		5%–20% degradation in at least one stressed condition	Degradation: Yes (Thermal and acid), Interference: No
		Resolution ≥ 2.0	Resolution: N/A
		Purity flag: No	Purity flag: No

#### 2.4.2 Assessment of chemical stability of ketoprofen

The UPLC method was modified for the assessment of the chemical stability of ketoprofen in the compounded topical formulations. The reverse phase, gradient method utilized a mobile phase A of 0.1% trifluoroacetic acid in purified water, and a mobile phase B of 0.1% trifluoroacetic acid in acetonitrile. The same 1.7 μm Acquity UPLC BEH C18 column used in the in vitro permeation study was selected. The column was heated to 50°C and samples were kept at 8°C. The flow rate was set at 0.8 mL/min. The composition of the mobile phase began with 75% A and 25% B, and it was gradually changed to 55% A and 45% B from 0 to 3 min. At 3.1 min, the gradient returned to the initial composition of 75% A and 25% B and remained the same until the end of the run at 4 min. A total of five calibration standards ranging from 50 μg/mL to 75 μg/mL were prepared to quantitate the amount of ketoprofen in the extracted sample solution.

Each compounded sample (0.5 g) was mixed with 39.5 mL methanol using a vortex and sonicator. The mixture was centrifuged at 6000 rpm for 10 min at room temperature and the resulting supernatant containing ketoprofen was diluted with methanol and micro‐centrifuged at 14,000 rpm for 10 min. A final supernatant at 62.5 μg/mL was analyzed by UPLC with the validated assay method. A 5‐point calibration curve of the ketoprofen peak area obtained from standard solutions was used for quantification by linear regression.

#### 2.4.3 Stability assay

One batch of 500 g was prepared and evenly distributed into 20 plastic jars with 25 g of gel per container. Ten jars were stored in a stability chamber at 5°C, and another 10 jars were stored in a stability chamber at 25°C with a relative humidity of 60%. Immediately after preparation, at 7, 14, 28, 42, 61, 91, 119, and 181 days, one unopened jar of ketoprofen compounded topical formulation was withdrawn from the stability chambers and analyzed for changes in color/appearance, and assay. Ketoprofen stability was determined by calculating the recovery percentage of the labeled concentration at each time interval. The mean and standard deviation from the three samplings were calculated and stability was defined as the retention of 90%–110% of the labeled concentration.

## 3 RESULTS

### 3.1 Evaluation of the cytotoxicity of the anhydrous gel

**FIGURE 1:**
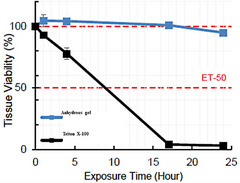
EpiDerm tissue viability profile after 24 h‐exposure to the anhydrous gel and 1% Triton X‐100. Viability was determined using the MTT assay.

Topical compounding bases should be non‐irritating and non‐toxic to minimize adverse effects and patient discomfort. The analysis of the cytotoxicity of the anhydrous gel on the skin model demonstrated that its application to EpiDerm did not affect cell proliferation after 17 h of exposure (Figure 1). A minimal decrease in cell viability (∼6% cell death) was observed after 24 h; in contrast, exposure of the skin tissue to Triton X‐100 1% (considered as a moderate‐to‐mild skin irritant) for 17 h caused ∼97% cell death (Figure 1). Triton X‐100 has been previously used as positive control in similar studies.^8^ The obtained ET50 value longer than 24 h for the anhydrous gel indicated that the gel can be classified as non‐irritating and equivalent to baby shampoo, as suggested by MatTek Corporation.^12^, ^13^

**FIGURE 2:**
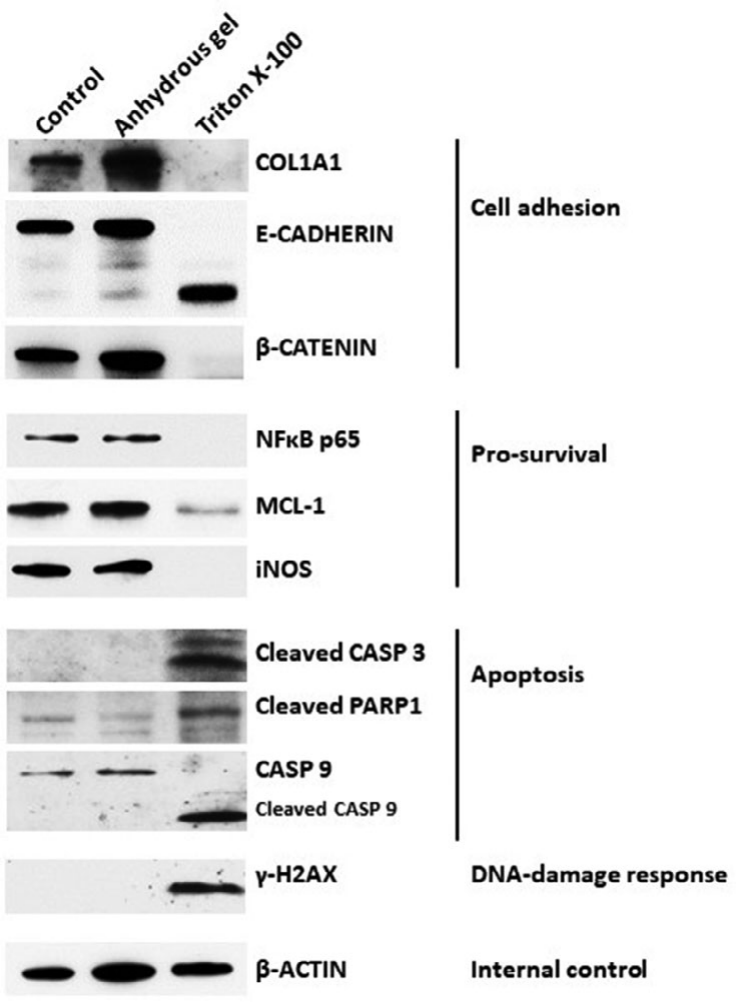
Western blot analysis of human skin cells exposed to the anhydrous gel and Triton X‐100. EpiDerm tissues were exposed to either the anhydrous gel or Triton X‐100 for 17 h and homogenized. Total protein concentration was determined, and cell lysates were analyzed by Western blotting.

The results of the MTT assay were consistent with changes in the level of cell adhesion molecules, which are important in cell proliferation and maintenance of skin integrity. Western blot analysis after exposure of EpiDerm samples to the anhydrous gel for 17 h showed minimal changes in the levels of Collagen Type 1 (COL1A1) and E‐CADHERIN. In contrast, Triton X‐100 obliterated the level of these two proteins; a faster‐moving fragment was identified by the anti‐E‐CADHERIN antibody (Figure 2) presumably a cleavage product of E‐CADHERIN, which is known to be proteolytically cleaved.^14^, ^15^ E‐CADHERIN is known to interact with β‐CATENIN and together with other proteins they form complex adherens junctions that provide a strong adhesive interface between cells to ensure proper skin function.^16^ Like E‐CADHERIN, β‐CATENIN almost disappeared in EpiDerm treated with Triton X‐100 but not with the anhydrous gel (Figure 2), suggesting maintenance of the integrity of the skin tissue exposed to the anhydrous gel.

The effects of the anhydrous gel on cell survival markers were then analyzed. Exposure of EpiDerm to the anhydrous gel for 17 h did not significantly affect the protein level of NFκB p65, which is a known proliferation‐related protein.^17^ In contrast, exposure of EpiDerm to Triton X‐100 significantly decreased the level of NFκB p65 and pro‐survival protein MCL‐1 (Figure 2). The expression of inducible Nitric Oxide Synthase (iNOS), a protein important for keratinocytes proliferation and epidermal permeability barrier homeostasis,^18^ was obliterated in tissues exposed to Triton X‐100 for 17 h but it did not change when the anhydrous gel was used (Figure 2).

To confirm if the anhydrous gel activated apoptosis, changes in the level and modification of proteins considered as death markers were determined. CASPASE 3 (CASP 3), considered to be an executioner of apoptosis,^19^ was activated by cleavage in EpiDerm exposed to Triton X‐100 for 17 h; its cleavage was not observed in the anhydrous gel‐treated tissue (Figure 2). This cleavage of CASP3 is known to lead to cleavage of PARP1,^20^ and as expected, Triton X‐100 significantly induced cleavage of PARP1 after 17 h (Figure 2). Like CASP 3, CASPASE 9 (CASP 9) is another executioner of apoptosis which is activated by cleavage.^21^ Exposure of the EpiDerm tissue to Triton X‐100 resulted in significant cleavage of CASP 9, which was not observed in the anhydrous gel‐treated tissue (Figure 2).

The observed activation of caspases may lead to cleavage of genomic DNA and concomitant DNA‐damage response. In fact, the level of phosphorylated histone 2AX (γ‐H2AX), a known marker of DNA‐damage response,^22^ increased in EpiDerm tissue treated with Triton X‐100 but not with the anhydrous gel (Figure 2). Overall, the lack of deleterious effects of the anhydrous gel on the skin integrity, cell proliferation/survival, and genomic DNA damage suggests its safety when in contact with human skin.

### 3.2 In vitro permeation test of ketoprofen compounded topical formulations

**FIGURE 3:**
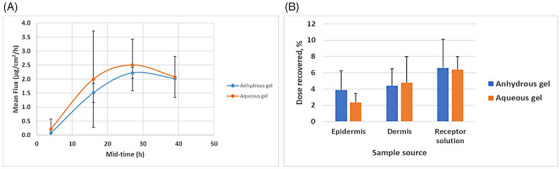
Comparison of skin absorption of ketoprofen in the anhydrous and aqueous gels. (A) The mean flux rate of ketoprofen in two compounded formulations were determined as described under “Materials and Methods.” (B) The distribution of ketoprofen across different parts of the skin was also determined. Ketoprofen in either the anhydrous gel or aqueous gel was applied onto three donated abdomen skin samples and the distribution was determined after 48 h. Recovery was calculated based on the total amount of ketoprofen recovered per chamber.

The percutaneous absorptions of ketoprofen in the anhydrous and aqueous gel formulations were compared using three donors of human skin samples. A mean flux rate of 0.072 and 0.217 μg ketoprofen/cm^2^/h in the anhydrous gel and the aqueous gel, respectively, was observed after 4 h of exposure (Figure 3A). These values increased to 2.224 and 2.505 μg ketoprofen/cm^2^/h in the anhydrous gel and aqueous gel, respectively, after 27 h of exposure, and showed a decreasing trend thereafter (Figure 3A). Statistical analysis did not show significant difference between the two formulations (data not shown).

While the absorption results indicate the rate of percutaneous absorption (mean flux) of ketoprofen through the skin (Figure 3A), the distribution results indicate the percutaneous absorption of ketoprofen into the skin. For the anhydrous gel, the amount of ketoprofen (i.e., the mass recovered after 48 h) within the epidermis and dermis was 73 ± 40 μg and 85 ± 35 μg, respectively. In contrast, the total ketoprofen recovery for the aqueous gel formulation was 44 ± 16 μg and 88 ± 52 μg within the epidermis and dermis, respectively. These results are presented in Figure 3B as dose recovered, which was calculated based on the total amount of ketoprofen recovered per chamber. Despite the apparent differences of percutaneous absorption of ketoprofen between the two topical compounded formulations, these differences were not statistically significant.

### 3.3 Stability study of ketoprofen compounded topical formulations

The level of ketoprofen was quantified using a stability‐indicating UPLC assay method and, as shown in Table 3, all the criteria were met suggesting the validity of the UPLC method.

**FIGURE 4:**
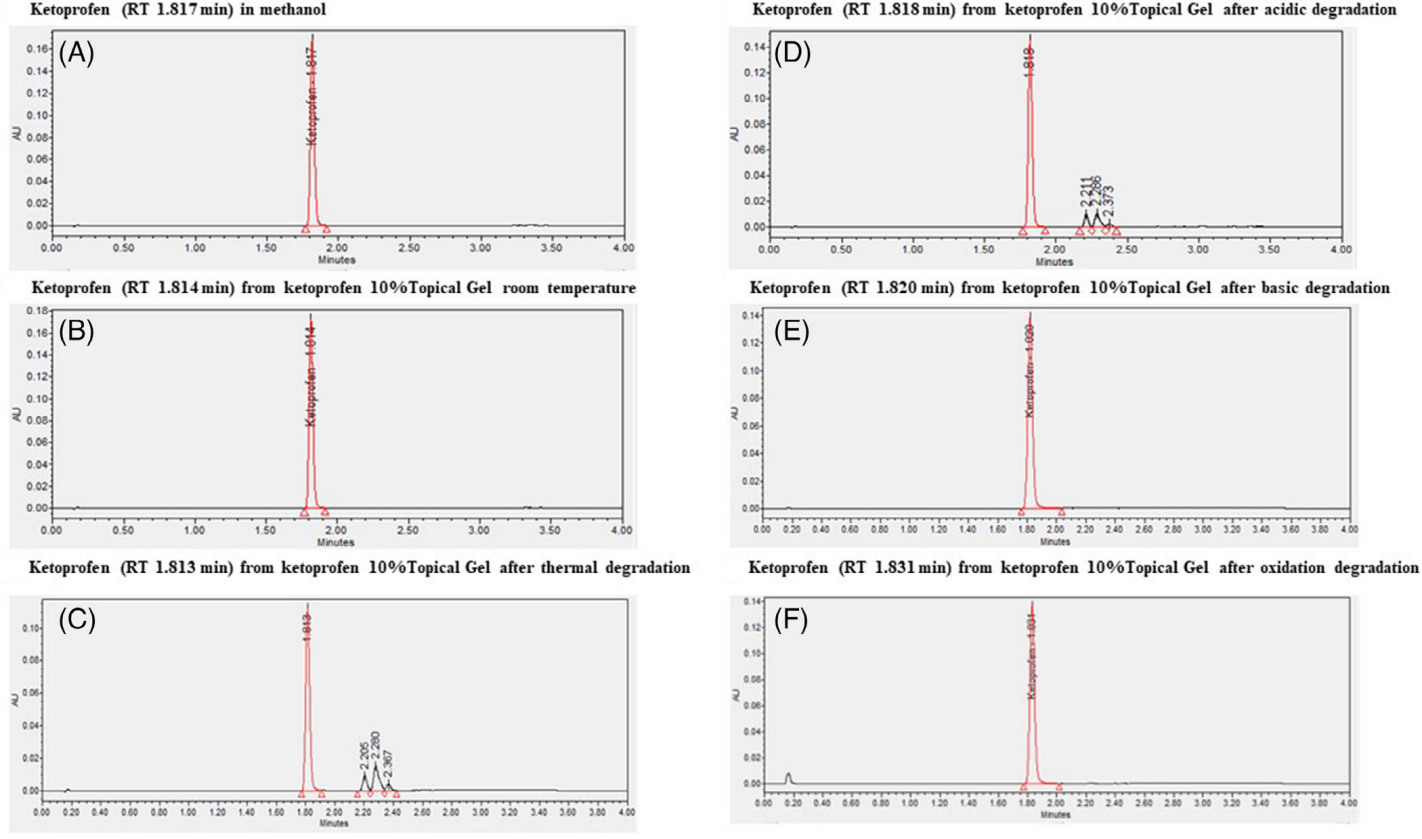
UPLC chromatograms of ketoprofen in the anhydrous gel. Ketoprofen was prepared in (A) methanol or extracted from compounded 10% ketoprofen in the anhydrous gel (B) kept at room temperature for 7 days, or after (C) thermal (60°C for 14 days), (D) acidic (0.1 N HCl for 7 days at room temperature), (E) basic (0.1 N NaOH for 7 days), and (F) oxidation (20% H_2_O_2_ for 7 days) degradation.

The efficiency of the UPLC method was further assessed by stressing the formulation of ketoprofen 10% in the anhydrous gel under different conditions, and separating ketoprofen from its degradation products. Ketoprofen prepared in methanol or extracted from its gel formulation had retention times (RT) of 1.817 and 1.814 min, respectively, without any degradation product detected (Figure 4A). A significant degradation (15.69%) occurred in the sample exposed to thermal degradation at 60°C for 14 days, with ketoprofen eluting at 1.813 min (Table 4 and Figure 4C). Under an acidic condition of 0.1 N HCl for 7 days, a mild degradation (7.58%) was observed (Table 4 and Figure 4D). The degradation products under hot and acidic conditions had retention times of 2.21, 2.29, and 2.37 min, respectively, which were well‐separated from the ketoprofen peak. No significant degradation of ketoprofen was observed under basic and oxidation conditions (Figure 4E,F). The results show absence of peak interfering with the ketoprofen peak, and the UPLC method demonstrated specificity for the ketoprofen assay in the presence of components likely to be generated during long‐term storage of the preparation.

**TABLE 4:** Degradation of ketoprofen topical gel under stressed conditions.

Degradation condition	Initial potency (%)	Final potency (%)	Degradation (%)	Tailing factor	Resolution	Purity flag
Thermal	99.35%	83.76%	–15.69%	1.14	N/A	No
Acid	103.21%	95.39%	–7.58%	1.14	N/A	No
Base	103.21%	99.77%	–3.33%	1.14	N/A	No
Oxidation	103.21%	98.48%	–4.58%	1.14	N/A	No

**FIGURE 5:**
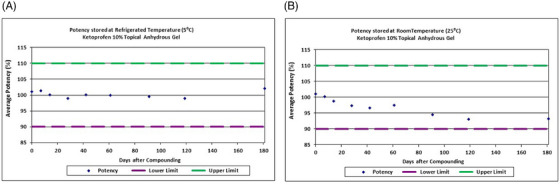
Stability of 10% ketoprofen in the anhydrous gel. The compounded formulation was stored at (A) refrigerated (5°C) and (B) room temperature (25°C) for 181 days and analyzed for the level of ketoprofen by UPLC. The dashed lines represent the specific range of 90.0% to 110.0% acceptable limits. The values are average of nine UPLC injections from three extractions.

Following the validity of the UPLC method, the chemical stability was determined for ketoprofen 10% in the anhydrous gel stored under refrigerated temperature (5°C) and controlled room temperature (25°C). On day 0, the mean potency of ketoprofen was 101% (equivalent to ketoprofen 10.1% in the anhydrous gel). The initial concentration of ketoprofen 10% was set as the baseline for percent remaining in the duration of the study. At refrigerated temperature, the percentage remaining of ketoprofen was within 98.9%–102.0% from day 7 to 181 days. At controlled room temperature, the percentage remaining of ketoprofen was within 93.1%–101.0% from day 7 to 181 days (Figure 5). By setting the limits at 90%–110% of the labeled concentration of ketoprofen, the results suggest that ketoprofen in the anhydrous gel is chemically stable within 181 days of storage under refrigerated or room temperature (Figure 5). The appearance and color of the compounded topical formulation remained an off‐white cream throughout the duration of the study in both storage conditions.

## 4 DISCUSSION

The complex interaction between the biological and mechanical properties of a compounding base for transdermal delivery and the physicochemical characteristics of the drug(s) incorporated on the base is a critical factor that contributes to the efficiency of drug delivery and drug stability. In this study, a proprietary anhydrous gel was evaluated as far as safety and percutaneous delivery of ketoprofen was concerned.

The lack of cytotoxicity of the anhydrous gel to human skin cells is supported by cellular and biochemical experimental results. Using the EpiDerm human skin tissue model, the ET50 value (exposure time with 50% cell survival) for the anhydrous gel was not reached after 24‐h exposure. The observed ET50 value of more than 24 h is above the skin irritation threshold based on the classification described by MatTek Corporation^12^ and Song et al.,^13^ suggesting that the anhydrous gel can be classified non‐irritating.

This non‐irritating property of the anhydrous gel is consistent with the minimal change observed in the levels of molecular protein markers of cell adhesion, cell proliferation and apoptosis in cells exposed to the anhydrous gel (Figure 2). Specifically, the expression of cell adhesion markers including collagen COL1A1, E‐CADHERIN and β‐CATENIN was not affected. Collagen promotes the mesenchymal stem cells to repair the skin damage in terms of epidermal layer repair and cell proliferation^23^ whereas E‐CADHERIN and β‐CATENIN are important for epidermal barrier function by regulating tight skin cell junctions.^24^ Cleavage of E‐CADHERIN destabilizes the interactions between adjacent epithelial cells and contributes to loss of monolayer integrity,^14^ which was observed in Triton X‐100‐treated skin tissues but not in the anhydrous gel‐treated EpiDerm (Figure 2). Consistent with the maintenance of the integrity of the skin tissue, the status of pro‐survival proteins including NFκB p65, MCL‐1, and iNOS did not change in Epiderm exposed to the anhydrous gel. NFκB p65 is important for skin cell proliferation^25^, ^26^ and MCL‐1 is a pro‐survival protein which inhibits the functions of pro‐apoptotic proteins.^27^ The inducible NOS (iNOS) is another protein involved in keratinocyte proliferation by catalyzing the synthesis of nitric oxide.^18^ The results presented in this study show lack of change in the expression of NFκB p65, MCL‐1, and iNOS in EpiDerm exposed to the anhydrous gel (Figure 2) indicating that the gel does not inhibit skin cell proliferation. Moreover, the absence of effects on the cleavage of CASPASE 3, PARP1 and CASPASE 9 proteins is consistent with the safety of the anhydrous gel.

After showing the safety of the anhydrous gel on skin cells, its possible application on the delivery of ketoprofen was determined. The percutaneous permeation mean flux rate for ketoprofen in three donated human skin samples peaked ∼2.2 μg/cm^2^/h after 27 h, and started to decline and reached ∼ 2 μg/cm^2^/h after 39 h (Figure 3A). The permeation of ketoprofen in the aqueous gel was not significantly different. The formulation of ketoprofen in the anhydrous gel was found stable at 5°C and 25°C within 181 days (Figure 5). The anhydrous nature of the proprietary gel and its stability within 181 days suggest that APIs incorporated in this anhydrous gel may have longer BUD when compared to aqueous gel bases.

This study has few limitations that should be taken into consideration. Higher number of donated human skin samples could have increased the statistical power and more significant results could have been obtained. The study is an in vitro evaluation which may only be considered a prediction of the in vivo skin absorption.^28^ Moreover, the anatomical sites and ages of the donors may affect the percutaneous absorption of ketoprofen.^29^ Due to these limitations, the findings in this study are considered preliminary until a more comprehensive analysis is performed. Nevertheless, the results presented in this study provide the pharmacy compounding industry an option for the percutaneous delivery of ketoprofen compounded topical formulations.

## 5 CONCLUSION

The proprietary anhydrous gel discussed in this study is safe to use as a compounding base for transdermal delivery of ketoprofen. Although the anhydrous and aqueous gels described can deliver similar amounts of ketoprofen, the anhydrous gel compounded topical formulation provides extended stability and, therefore, longer default BUD.

## CONFLICT OF INTEREST STATEMENT

The authors K. Ip, G. Song, D. Banov, A. Bassani, Y. Liu, and H. Song are employees of PCCA, the manufacturer of the proprietary anhydrous and aqueous bases.

## ETHICS STATEMENT

Skin samples were purchased from Genoskin (Salem, MA) and the company obtained informed consent from the donors and the ethics for tissue collection was approved by the French Minister of Research (No. AC‐2022‐4863).

## Data Availability

The data that support the findings of this study are available from the corresponding author upon request.
